# Carbohydrate Mouth Rinse Maintains Muscle Electromyographic Activity and Increases Time to Exhaustion during Moderate but not High-Intensity Cycling Exercise

**DOI:** 10.3390/nu8030049

**Published:** 2016-03-09

**Authors:** Victor José Bastos-Silva, Alan de Albuquerque Melo, Adriano Eduardo Lima-Silva, Felipe Arruda Moura, Rômulo Bertuzzi, Gustavo Gomes de Araujo

**Affiliations:** 1 Research Group Applied to Sport Science—GPCAE, Federal University of Alagoas, Avenue Lourival Melo Mota, s/n, Maceió-AL 57072-900, Brazil; victormat16@hotmail.com (V.J.B.-S.); alan_melo@hotmail.com (A.A.M.); 2 Sport Science Research Group—GPCE, Federal University of Pernambuco, Alto do Reservatório street, Bela Vista, Vitória de Santo Antão-PE 608-680, Brazil; adrianosilva@usp.br; 3 Laboratory of Applied Biomechanics, State University of Londrina, Avenue Gil de Abreu e Souza, 2335, Unidade 1121, Esperança, Londrina 86058-100, Brazil; felipearrudamoura@gmail.com; 4 Endurance Performance Research Group, University of São Paulo, Avenue Prof. Mello de Morais, 65, São Paulo SP 05508-030, Brazil; bertuzzi@usp.br

**Keywords:** athletic performance, central nervous system, maltodextrin, mouthwash

## Abstract

The aim was to investigate the influence of a carbohydrate (CHO) mouth rinse on the vastus lateralis (VL) and rectus femoris (RF) electromyographic activity (EMG) and time to exhaustion (TE) during moderate (MIE) and high-intensity cycling exercise (HIE). Thirteen participants cycled at 80% of their respiratory compensation point and at 110% of their peak power output to the point of exhaustion. Before the trials and every 15 min during MIE, participants rinsed with the CHO or Placebo (PLA) solutions. The root mean square was calculated. CHO had no effect on the TE during HIE (CHO: 177.3 ± 42.2 s; PLA: 163.0 ± 26.7 s, *p* = 0.10), but the TE was increased during MIE (CHO: 76.6 ± 19.7 min; PLA: 65.4 ± 15.2 min; *p* = 0.01). The EMG activity in the VL was higher than PLA at 30 min (CHO: 10.5% ± 2.6%; PLA: 7.7% ± 3.3%; *p* = 0.01) and before exhaustion (CHO: 10.3% ± 2.5%; PLA: 8.0% ± 2.9%; *p* = 0.01) with CHO rinsing. There was no CHO effect on the EMG activity of RF during MIE or for VL and RF during HIE. CHO mouth rinse maintains EMG activity and enhances performance for MIE but not for HIE.

## 1. Introduction

The effects of carbohydrate (CHO) ingestion before and during prolonged exercise (>60 min) are well documented in the literature [1,2,3]. CHO intake is beneficial during prolonged exercise because it maintains blood glucose levels and the CHO oxidation rate [1]. However, these mechanisms cannot explain the ergogenic effect of CHO intake for events lasting an hour or less [4], because exogenous CHO oxidation minimally contributes to the total CHO oxidation in the muscle during this type of exercise [5].

Accordingly, the ergogenic effect of CHO intake during exercise lasting an hour or less has been assigned to a direct effect of CHO on the central nervous system, which is possibly mediated by CHO receptors located in the mouth. In this regard, Carter *et al.* [6] investigated the effect of a CHO mouth rinse during a ~60 min cycling time trial and found that CHO mouth rinse improves performance by ~3% compared to placebo mouth rinse (PLA). Other authors confirmed the ergogenic effect of CHO mouth rinse during efforts lasting approximately 60 min [7,8,9]. A possible explanation for the positive effect of CHO mouth rinse on performance is related to activation of the brain areas associated with motor control, reward and pleasure, which may cause a decrease in the rate of perceived exertion (RPE) during exercise [8,10]. Furthermore, it has been reported that CHO mouth rinse increases the excitability of the corticomotor pathway [11] and attenuates neuromuscular fatigue induced by exercise [12].

While the effect of CHO mouth rinse during 60 min of exercise (moderate-intensity) is well documented, little is known about its effect on supramaximal exercises (high-intensity). Moreover, it has not been demonstrated that CHO mouth rinse is able to change the neuromuscular stimulation in cyclical activities, even cyclical activities which are reported to be the most susceptible to the effects of CHO mouth rinse [6,7,8,9].

Therefore, the aim of this study was to investigate the influence of CHO mouth rinse on muscle activation (as inferred by electromyography, EMG) and time to exhaustion (TE) during moderate- and high-intensity exercise. We hypothesized that CHO mouth rinse would be able to maintain muscle recruitment and improve TE at both intensities.

## 2. Materials and Methods

### 2.1. Participants

Thirteen physically active (minimum of 150 min·week^−1^ of moderate exercise) healthy men (age = 23.1 ± 2.6 years; body mass = 74.7 ± 10.9 kg; height = 175 ± 6 cm; body fat = 14.2% ± 6.8%; VO_2max_ = 41.6 ± 7.6 mL/kg/min; peak power output (PPO) = 252.3 ± 36.3 W) volunteered to participate in the present study. Participants were provided with information about the experimental risks and signed an informed consent form before starting the experiments. The study procedures were conducted in accordance with the Declaration of Helsinki (2008) and were approved by the local ethics committee (protocol number: 16573413.8.0000.5013).

### 2.2. Experimental Protocol

The study was conducted in a crossover, counterbalanced, and double blind model. Five visits were performed with a minimum of 72 and maximum of 96 h intervals. In the first session, anthropometric measurements (body weight, height, and body fat percentage) and maximal incremental exercise tests to measure the VO_2max_ and PPO corresponding to VO_2max_ were performed. During the following four visits, participants performed high-intensity exercise at 110% of PPO or moderate-intensity exercise at 80% of respiratory compensation point (RCP) after washing their mouths with CHO or PLA. All tests were performed at the same time of day, two hours after the last meal [13]. Participants were instructed to repeat their food intake in the 24 h prior to each experimental trial. Participants refrained from exhaustive exercise, alcohol, caffeine, or any nutritional supplements 48 h before each trial.

### 2.3. Anthropometric Measurements

Body weight was obtained using an electronic scale, and height with a stadiometer. The skinfold thickness was measured with a Lange caliper. The body density was predicted using the generalized equation of Jackson & Pollock [14], and body fat was estimated using the equation of Siri [15].

### 2.4. Maximal Incremental Exercise Test

The maximal incremental exercise test was performed on an electromagnetically braked cycle ergometer (Ergo Fit 167, Ergo-FitGmbH & Co., Pirmasens, Germany). The seat height was adjusted for each participant, allowing near-full leg extension during each pedal revolution, and these conditions were reproduced for all experimental sessions. After a 3 min warm-up at 30 W, the power output was increased 30 W·min^−1^ while maintaining pedal frequency at 60–70 rpm until exhaustion, which was defined as the incapacity to maintain a minimum pedal cadence of 60 rpm. The participants received strong verbal encouragement to continue for as long as possible.

Oxygen uptake (VO_2_) was measured breath-by-breath throughout the test using a gas analyser (Quark, Cosmed, Rome, Italy) and averaged over 30 s intervals. The device was calibrated according to the manufacturer’s specifications using ambient air, gas containing 20.9% O_2_ and 5% CO_2_, and a 3 L syringe. The VO_2max_ was determined when the following criteria were met: an increase in the VO_2_ of less than 2.1 mL·kg^−1^·min^−1^ on two consecutive stages and a respiratory exchange ratio greater than 1.1 and ±10 bpm of the maximal age-predicted heart rate [16]. PPO was considered as the maximal power output reached in the trial. The RCP was visually identified by two experienced evaluators using a cluster of parameters, *i.e.*, the second break at the ventilation curve, an increase in the VE/VCO_2_ ratio, and the first fall point of the CO_2_ fraction [17].

### 2.5. Moderate and High-Intensity Exercises

After 4 min of warm-up at 30 W, the workload was adjusted to a power output corresponding to 110% of PPO for high-intensity exercise or to 80% of RCP for moderate-intensity exercise. Both tests were interrupted when the pedal cadence was maintained below 60 rpm for more than 5 s or three times consecutively.

### 2.6. Mouth Rinse Protocol

During both tests, the participants rinsed their mouths with CHO (25 mL solution with 6.4% maltodextrin) or PLA (25 mL solution without CHO). Both solutions had the same flavour (orange), smell and density. The participants made movements with their tongues, keeping the solution in their mouths for 10 s, and then expelled the solution into a container [18]. The participants did not report being able to distinguish a difference between the CHO and PLA conditions. The solutions were administered immediately before and every 15 min until exhaustion during the moderate-intensity exercise or only immediately before in the high-intensity exercise.

### 2.7. Rating of Perceived Exertion (RPE)

The perceived exertion was recorded before and at the end of the high-intensity exercise or before, every 15 min, and at the end of moderate-intensity exercise. The Borg Scale (6–20) was used [19].

## 3. Acquisition and Analysis of the EMG Signal

The EMG signals of the vastus lateralis (VL) and rectus femoris (RF) of the right quadriceps were captured at 2000 Hz using an electromyography system (model 410c, EMG system Brazil, São Paulo, Brazil). Initially, shaving was followed by asepsis with alcohol, and was performed to reduce skin impedance. Then, bipolar surface electrodes (Ag/AgCl) were placed on the VL and RF muscles. The reference electrode was positioned at a neutral location (tibia). The electrodes were placed on the skin using adhesive tape to minimize wire movement. The placement and location of the electrodes were in accordance with the recommendations of Hermens *et al.* [20].

In high-intensity exercise, the EMG signal was captured throughout the test. In moderate-intensity exercise, the EMG acquisition was captured during 3 min at the after each oral rinse. The signal was filtered using a Butterworth filter 3rd order and cut-off frequencies of 20 and 450 Hz. The root mean square (RMS) was used as an indicator of total muscle activation [21]. In the high-intensity exercise, RMS was normalized to the average of the last 30 s of the raw signal. In the moderate-intensity exercise, the RMS was normalized by its maximum raw signal obtained throughout the test. The RMS was analysed during 3 min periods for moderate-intensity exercise (*i.e*., 0–3 min, 15–18 min, 30–33 min and the last 3 min before exhaustion) and during 30 s periods for high-intensity exercise (*i.e*., 0–30 s, 30–60 s, 60–90 s, 90–120 s, and the last 30 s before exhaustion).

### 3.1. Statistical Analysis

The data distribution was evaluated for normality with the Kolgomorov-Smirnov test. A paired *t*-test was used to examine the differences between the CHO and PLA conditions for TE. The RMS was analysed using a two-way repeated measures ANOVA, which was followed by Fisher *post hoc* tests (normal distribution). The RPE between the CHO and PLA conditions was compared using the Wilcoxon test. All statistical tests were performed using SPSS software (version 13.0, Chicago, IL, USA) with a significance level lower than 5% (*p* < 0.05).

### 3.2. Results

**Figure 1:**
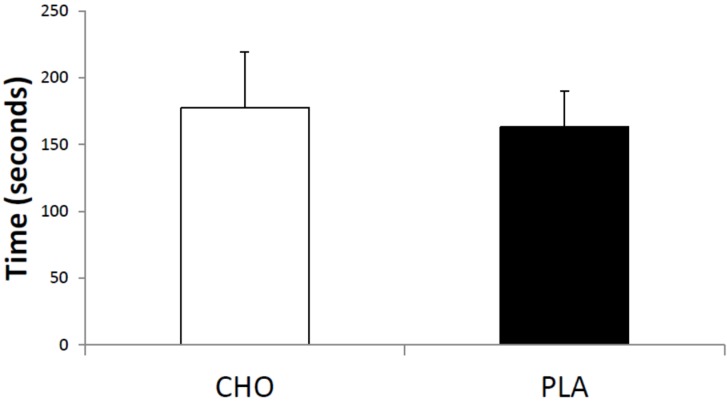
Time (seconds) to exhaustion during high-intensity exercise. CHO = carbohydrate; and PLA = placebo.

**Figure 2:**
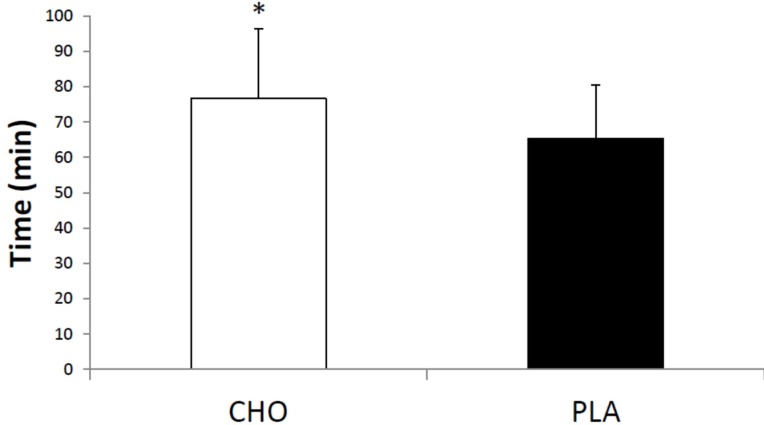
Time (min) to exhaustion during moderate-intensity exercise. CHO = carbohydrate and PLA = placebo. * Different from PLA, *p* < 0.05.

The power outputs used for high-intensity and moderate-intensity exercise were 276.1 ± 41.2 W and 155.3 ± 26.8 W, respectively. There was no significant difference in the TE between the CHO and PLA conditions for high-intensity exercise (CHO: 177.3 ± 42.2 s and PLA: 163.0 ± 26.7 s, *p* = 0.10, Figure 1). However, the TE was significantly longer (+14%) for CHO than for PLA during moderate-intensity exercise (CHO: 76.6 ± 19.7 min and PLA: 65.4 ± 15.2 min; *p* = 0.01, Figure 2).

**Figure 3:**
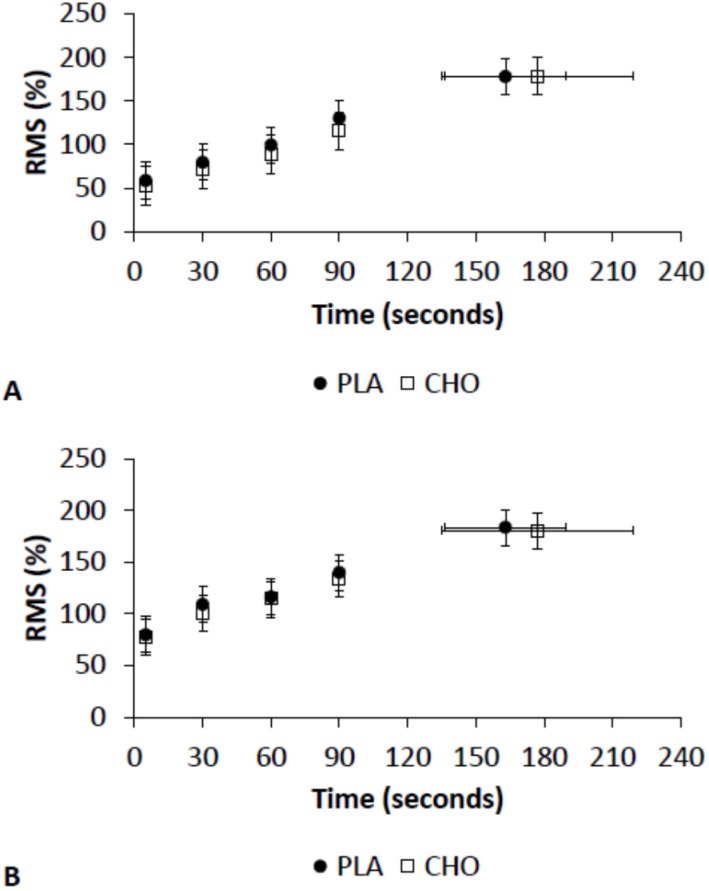
EMG activity during high-intensity exercise in (**A**) rectus femoris (RF); and (**B**) vastus lateralis (VL). Root mean square (RMS) was used as an indicator of total muscle activation.

**Figure 4:**
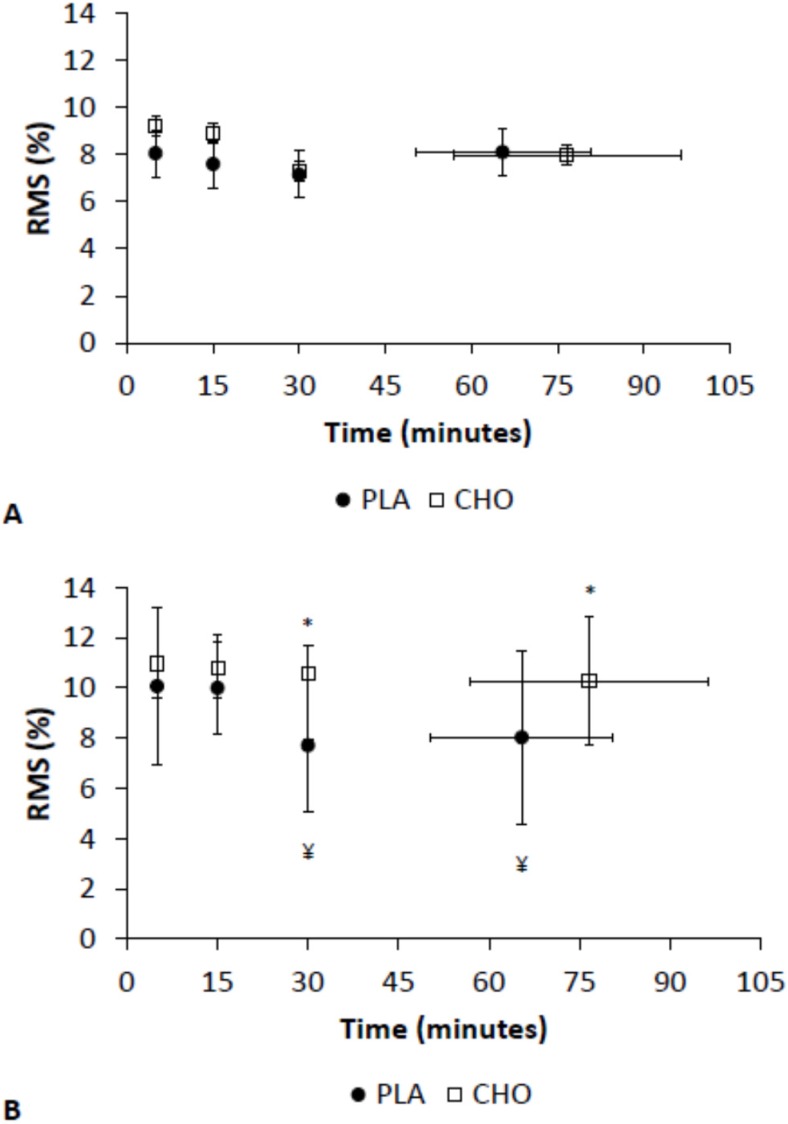
EMG activity during moderate-intensity exercise in (**A**) rectus femoris (RF); and (**B**) vastus lateralis (VL). * Different from PLA, *p* < 0.01. ¥ Different from time 0 and 15 for placebo only, *p* < 0.03.

There were no significant effects of CHO mouth rinse on the RF and VL EMG activity during high-intensity exercise (Figure 3A,B). However, the EMG activity of the VL muscle was higher at 30 min and at exhaustion for CHO than for PLA during moderate-intensity exercise (Figure 4B). There was no effect of CHO mouth rinse on the EMG activity of the RF muscle during moderate-intensity exercise (Figure 4A).

CHO mouth rinse also reduced the RPE at 15 min during moderate-intensity exercise (CHO = 10.9 ± 2.3 and PLA = 12.3 ± 2.9 units; *p* = 0.04) and at exhaustion during the high-intensity exercise (CHO = 18.2 ± 1.1 and PLA = 19.2 ± 1.0; *p* = 0.01). There was no difference for the other time points at any of the exercise intensities.

## 4. Discussion

Our main findings were as follows: (1) increase in the TE for moderate-intensity; (2) VL EMG maintenance for moderate-intensity; (3) reduction of the RPE in a 15 min moderate-intensity trial and (4) reduction of the RPE in exhaustion during the high-intensity trial. However, the CHO mouth rinse during high-intensity exercise did not improve performance or muscle activity.

Regarding the action of the CHO mouth rinse on the motor cortex, Gant *et al.* [11] reported increased corticomotor excitability during isometric exercise of elbow flexion with CHO mouth rinse. Moreover, these authors reported that CHO mouth rinse may have a greater influence on the excitability of the corticomotor pathway during fatigue, where CHO increased the motor evoked potentials amplitude by 9% in fresh muscle and 30% in fatigued muscle. Ours is the first report on the effect of CHO mouth rinse on the EMG signal during whole-body exercise. We observed a reduction in the EMG during moderate-intensity exercise over time for the PLA condition. However, the EMG activity in the CHO mouth rinse was maintained intra-group, but was greater than for PLA at 30 min and at exhaustion, suggesting that the effect of CHO mouth rinse is higher in fatigued muscle. In addition, Jeffreys *et al.* [12] suggest that CHO mouth rinse may attenuate the reduction in neuromuscular fatigue that occurs during cycling. However, the CHO mouth rinse seems to act on only the VL muscle. It is interesting to note that the VL is the most recruited muscle during cycling races [22].

Another possible factor explaining the longer TE during moderate-intensity exercise in the CHO condition was the reduced RPE in the middle of the trial. Studies [6,7,8,9] with CHO mouth rinse in a time trial reported no difference in the RPE. The RPE during time trial could be interpreted as a positive effect because the power output was increased with CHO mouth rinse.

However, these mechanisms were not replicated during high-intensity exercise (<5 min), as evidenced by TE and RMS between CHO and PLA mouth rinse. Additionally, other studies on CHO mouth rinse and power exercises [23], field testing with multiple sprints [24], or maximum strength [25], did not find beneficial effects. One possible explanation is that there is no ergogenic effect of CHO mouth rinse on high-intensity exercise; as a result, the improvement in the feeling of reward/pleasure is the only factor reducing the RPE, as observed in our study. However, this mechanism is insufficient to improve performance. Moreover, another factor is that during supramaximal tests, the recruitment of motor drive may be close to the maximum [26] such that no additional effect of CHO mouth rinse on muscle recruitment is possible.

## 5. Conclusions

CHO mouth rinse improves endurance time during moderate- but not high-intensity exercise, which is accompanied by maintenance of EMG activity and lower RPE. CHO mouth rinsing protocol can be applied to optimize moderate intensity training protocols, improving performance and delaying the onset of muscle fatigue.
